# Learning the Biochemical Basis of Axonal Guidance: Using *Caenorhabditis elegans* as a Model

**DOI:** 10.3390/biomedicines11061731

**Published:** 2023-06-16

**Authors:** Andreia Teixeira-Castro, João Carlos Sousa, Cármen Vieira, Joana Pereira-Sousa, Daniela Vilasboas-Campos, Fernanda Marques, Perpétua Pinto-do-Ó, Patrícia Maciel

**Affiliations:** 1 Life and Health Sciences Research Institute (ICVS), School of Medicine, University of Minho, Campus de Gualtar, 4710-057 Braga, Portugal; 2 ICVS/3B’s-PT Government Associate Laboratory, 4806-909 Guimarães, Portugal; 3 Instituto de Investigação e Inovação em Saúde (i3S), Universidade do Porto, 4200-135 Porto, Portugal

**Keywords:** neuronal cell biology, axon pathfinding, growth cone, molecular cues, experimental activity

## Abstract

Aim: Experimental models are a powerful aid in visualizing molecular phenomena. This work reports how the worm *Caenorhabditis elegans* (*C. elegans*) can be effectively explored for students to learn how molecular cues dramatically condition axonal guidance and define nervous system structure and behavior at the organism level. Summary of work: A loosely oriented observational activity preceded detailed discussions on molecules implied in axonal migration. *C. elegans* mutants were used to introduce second-year medical students to the deleterious effects of gene malfunctioning in neuron response to extracellular biochemical cues and to establish links between molecular function, nervous system structure, and animal behavior. Students observed *C. elegans* cultures and associated animal behavior alterations with the lack of function of specific axon guidance molecules (the soluble cue netrin/UNC-6 or two receptors, DCC/UNC-40 and UNC-5H). Microscopical observations of these strains, in combination with pan-neuronal GFP expression, allowed optimal visualization of severely affected neurons. Once the list of mutated genes in each strain was displayed, students could also relate abnormal patterns in axon migration/ventral and dorsal nerve cord neuron formation in *C. elegans* with mutated molecular components homologous to those in humans. Summary of results: Students rated the importance and effectiveness of the activity very highly. Ninety-three percent found it helpful to grasp human axonal migration, and all students were surprised with the power of the model in helping to visualize the phenomenon.

## 1. Introduction

**Figure 1:**
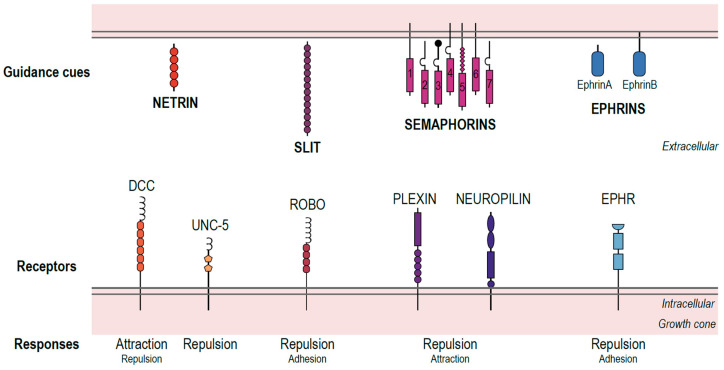
Summary of the four conserved families of guidance cues that determine neuronal and axonal migration and their respective receptors. Some guidance cues, such as Netrins, Slits, and a few semaphorins, are secreted to the extracellular media, while the majority of semaphorins and ephrins are expressed at the target cell surface. Each of these guidance cues binds to specific receptors expressed at the growth cone of the traveling pioneer neuron/axon. For instance, Netrins bind to DCC or UNC-5 receptors, Slits to Robo receptors, Semaphorins to Plexin and Neuropilin, and Ephrins to Eph receptors. These interactions result in differential responses at the growth cone (figure showing the most common response and other responses). The seven classes of Semaphorins are represented. DCC—deleted in colorectal cancer, Robo—Roundabout.

The development of an adequately wired nervous system is essential for the survival of many animal species, including humans. This biological “construction” depends on a tightly regulated genetic program and involves multiple cell proliferation, differentiation, migration, and elimination steps. Wiring of the nervous system also includes extension, specification, and elongation of a particular type of cellular process—the axon—in specific directions, followed by establishing and eliminating synapses, the points of connection between neurons, and a point of significant plasticity even after development. An important component of the proper wiring of nervous systems is the guidance of axonal growth, achieved through the time- and space-specific expression of soluble and diffusible biochemical cues, adhesion molecules, extracellular matrix components, and receptors able to sense those cues and transduce signals to the interior of the axonal process, where cytoskeleton-modifying molecules produce either the advancement of the growth cone (generating an attraction effect) or its collapse (producing a repulsion movement) (reviewed in detail in [1,2,3]). A summary of the main types of molecular performers involved in axonal pathfinding is presented in Figure 1.

The dysfunction of any of the components of this system generates axonal pathfinding defects that can vary in severity and their functional impact. In more complex nervous systems, there is a significant degree of redundancy. Nevertheless, some genetic mutations affecting the most relevant performers in axon guidance will lead to detectable behavioral phenotypes and nervous system diseases. In humans, these include some autism spectrum disorders and other neurodevelopmental disorders (reviewed in detail in [4]), and more specific disorders, such as horizontal gaze palsy with progressive scoliosis, caused by mutations in the slit ROBO3 receptor (OMIM 607313; [5]), congenital mirror movements that are associated with DCC mutations (OMIM 157600; [6]), congenital fibrosis of the extraocular muscles type 3, and TUBB3 syndromes, related to mutations in TUBB3 (beta-tubulin III, a subunit of microtubules) (OMIM 600638 [7]). Axonal pathfinding disturbances are also increasingly thought to contribute to Alzheimer’s disease (AD) pathogenesis [8,9]; in particular, several axon guidance performers, such as netrin-1, ephA4 (a receptor of ephrins), and the semaphorin sema3A, are being unveiled as modulators (positive/negative) of AD pathogenesis, and prospective therapeutic targets.

Given the complexity of this field of study, it is rarely addressed in undergraduate Biology or Biochemistry programs or during the nervous system courses in medical student training. However, the topic provides a unique opportunity to establish links between nervous system anatomy, biochemistry, and genetics concepts, taking advantage of students’ curiosity towards this very intriguing scientific question. Here, we designed a student-centered activity, combining experimental and virtual components, to promote active learning of key concepts in axonal pathfinding.

**Figure 2:**
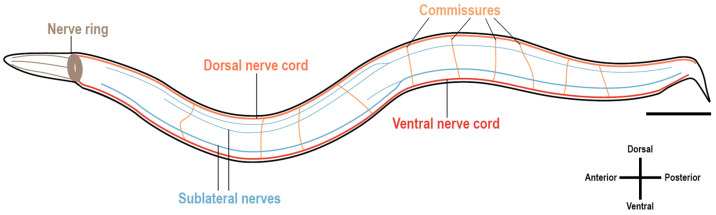
Simplified scheme of the *C. elegans* nervous system. The *C. elegans* nervous system is highly organized and comprises five components. The nerve ring is comprised of a bundle of head neurons, their axons, and processes; the ventral nerve cord is the major cord that spans longitudinally throughout the ventral midline of the animals and shelters the majority of peripheric neurons, such as the motor neurons; the dorsal nerve cord primarily consists of processes from the ventral cord motor neurons; commissures are circumferential tracts of neuronal processes spanning the dorsoventral axis and linking nerve cords; and the sublateral longitudinal nerves, which run under the body wall muscle cells and processes from some neurons join the sublateral cords. Scale bar: 100 µm.

We focused on three prototypic molecules: one soluble cue (netrin/UNC-6) with chemoattractant and chemorepulsive effects on two receptors (DCC/UNC-40 and UNC-5H, respectively). We used as a basis for the experimental activity the model organism *Caenorhabditis elegans* (*C. elegans*), a nematode for which the structure of the nervous system is very well characterized [10] (Figure 2) and easy to visualize with the aid of neuronally expressed fluorescent proteins given that the animal is transparent. The nervous system of *C. elegans* has 302 neurons categorized into at least 118 different neuronal classes, along with 56 glial cells providing neuronal support [11]. The connectome of *C. elegans* encompasses 4887 chemical synapses and 1447 gap junctions [10]. Furthermore, powerful genetic approaches have been used to dissect the mechanisms underlying axon guidance [12,13,14]. This allowed us, as a secondary aim, to convey the relevance of using model organisms for biomedical research with an impact on human health.

### Learning Activity Context and Structure

This learning activity was part of the University of Minho School of Medicine curriculum, within a three-unit block of curricular units named Organic and Functional Systems (I, II, and II), which integrated the contents of human Anatomy, Histology, Physiology, and Biochemistry, in an organic systems-based approach [15]. The activity was planned as part of a nervous system-focused module in the fourth semester of the medical course. Previous modules had included the core concepts of cell biology, molecular genetics, and biochemistry, and the key concepts in signal transduction necessary for the full grasping of the mechanisms of axonal pathfinding. Students were also experienced in using the microscope and had been trained in lab safety.

**Figure 3:**
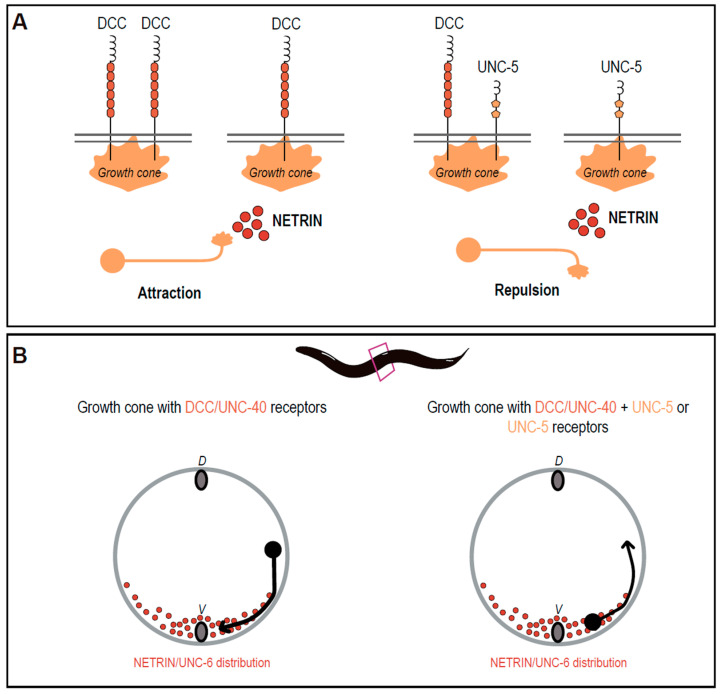
Netrin signaling through DCC/UNC-40 and UNC-5. (**A**) Long-range guidance cue netrin mediates attraction upon binding to DCC receptors and repulsion upon binding to UNC-5 or DCC/UNC-5 complex receptors at the growth cone. (**B**) In *C. elegans*, netrin/UNC-6 forms a distribution gradient at the ventral midline and is required for both ventral and dorsal projection of axons. Migrating growth cones expressing DCC/UNC-40 will primarily migrate ventrally, whereas those expressing DCC/UNC-40 and UNC-5 or UNC-5 will migrate dorsally. As *C. elegans* body structure resembles a circumference (in the represented coronal cut), dorsoventral migration also originates commissures formation.

The activity focused on three prototypical dorsal-ventral axon guidance-related molecules (Figure 3): netrin, deleted in colorectal cancer (DCC)/neogenin, and UNC5A-D (previously known as UNC-5H1-4) families. Netrins are evolutionarily conserved molecules that attract and repel axons to guide their growth. UNC-6, the sole *C. elegans* netrin ortholog, is expressed by ventral cells (neurons, glia, epidermoblasts, muscle, and vulva precursor cells) and provides positional information by establishing a ventral-dorsal concentration gradient [16,17,18]. Animals lacking UNC-6 function (*unc-6* mutant) are large and healthy but slightly fat and show a kinker motor phenotype. Regarding neuronal wiring, phenotypes include a disorganized ventral nerve cord (VNC), and dorsal extensions of DD and VD neurons that grow in aberrant directions, failing to reach the dorsal nerve cord (DNC) [19]. 

UNC-6 functions through two *C. elegans* transmembrane receptors: UNC-40 (mammalian DCC and neogenin) and UNC-5 (mammalian UNC5A-D). UNC-40 is the major conserved receptor involved in attraction to netrin and binds it directly [19,20]. UNC-40 is mainly required for ventral axon projections, causing a general failure of ventralward migrations, and variable defects in VD and DD commissures. *unc-40* mutant animals are weak kinker, dumpish, and slow but relatively active [21,22]. 

Repulsion from UNC-6/netrin primarily depends on the action of the UNC-5 transmembrane receptor, which also binds directly to netrin [23]. UNC-5 is required for dorsal projections. *unc-5* mutant animals demonstrate comparable growth to wild-type animals but exhibit severe motor defects (uncoordinated phenotype known as severe coiler), DNC is absent or almost absent, cord commissures fail to reach targets, and distribution of cell bodies of the VNC is disorganized. Direct interactions between UNC-40 and UNC-5 cytoplasmic domains are induced by binding to netrin. In the UNC-5/DCC receptor complex, the DCC protein potentiates the UNC-5 repulsive response [19,24,25].

Therefore, our rationale for the learning activity was to use guidance-related mutants, specifically *unc-6*, *unc-5,* and *unc-40*, which exhibit several motor and nervous system defects (Table 1 and Table 2). These phenotypes are a consequence of gene malfunctioning, resulting in impaired axon guidance.

The activity included the distribution of pre-class materials, namely a summary of the state of the art on the mechanisms of axonal pathfinding prepared by the teaching staff, two critical scientific articles for students to be able to go deeper into the subject if they so desired [18,26] and the activity guide. The lab activity lasted 90 min and was organized for classes of 45 students, each by 2 teachers. Students were organized into groups of three to five, each with access to a stereomicroscope. It started with a brief introduction of the work to be completed and of the *C. elegans* experimental model by the teacher (~10 min), including a brief overview of the structure of their nervous system. This was followed by the distribution of Petri dishes with the wild type (WT) and mutant nematode strains (named A to C) and of a score table to fill in (Table 1) with the morphological and behavioral phenotypes observed in these strains at the stereomicroscope (~25 min). Illustrative videos were recorded for each strain and are provided as supplementary material (Supplementary Video S1).

**Figure 4:**
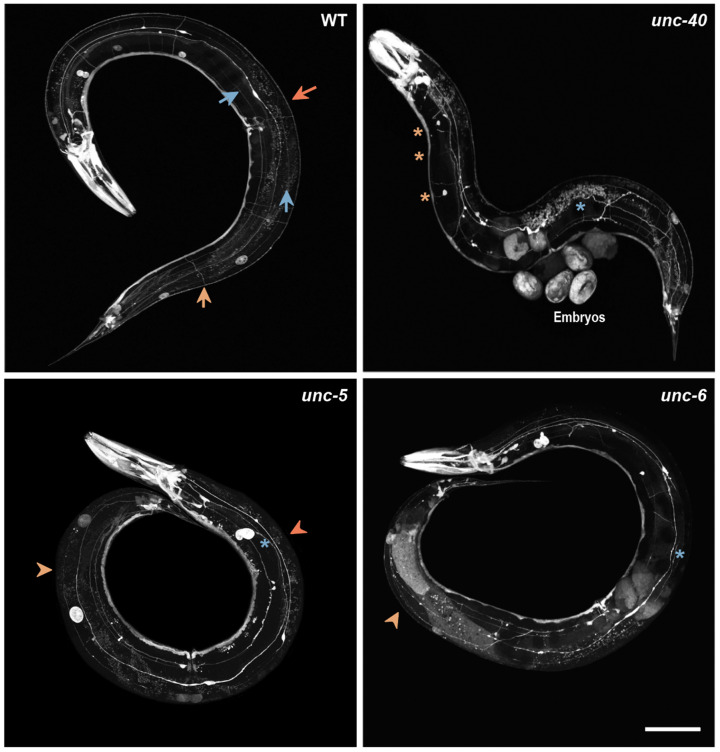
Confocal microscopy images of *C. elegans* nervous system, visualized via pan-neuronal expression of GFP (*P_unc-119_*::GFP). Wild-type animals show a complete and organized nervous system, while *unc-6*, *unc-5,* and *unc-40* mutant animals exhibit defects in axonal migration, most visible at the nerve cords, sublateral nerves, and commissures. Arrows in the WT panel indicate the presence and normal nervous system structures, while arrowheads in the mutant animals represent a lack of structures. Defective structures are labeled with an asterisk. Orange—DNC; yellow—commissures; and blue—sublateral nerves. Scale bar: 100 μm. WT—Wild-type; DNC—dorsal nerve cord.

Additionally, one unidentified strain (the “quiz strain”) was provided to the students. The students promptly said that it resembled WT animals. Then, the teacher explained (and showed using a fluorescence stereomicroscope) that the quiz strain expressed a transgene driving the expression of GFP to all *C. elegans* neuronal cells. Next, confocal microscopy images of the animals from each strain (wild-type and mutants) crossed with the quiz strain were projected and printed versions of the photos distributed to students (Figure 4), the aim being for each group to compare the structure of the nervous system between the wild-type and each mutant strain and register the observed differences (~20 min) (Table 2).

Finally, the identity of the mutants was revealed, and the groups (15 min), then the whole class (~20 min) discussed the possible mechanistic relationship between the absence of each specific guidance molecule or receptor and the observed phenotypes, considering their function(s).

Prompts for discussion were launched to the students. Some examples are given below:

1. What is the function of each of the molecules missing in each mutant strain?
2. Name the mammalian orthologue(s) of each molecule.
3. Name mediator(s) of attraction and repulsion signals.
4. Why do *unc-5* mutant animals have the DNC almost absent?
5. Why do *unc-40* mutant animals have VNC?

**Figure 5:**
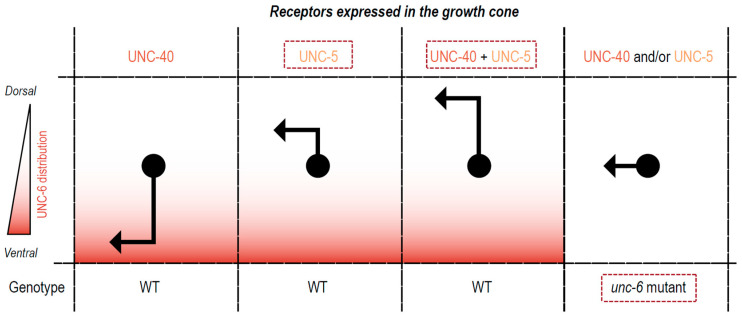
Summary of the model for specifying the dorsoventral positioning of longitudinal nerves in *C. elegans*. In WT animals, the ventrally biased gradient of UNC-6 modulates axonal migration by attracting growth cones expressing UNC-40 or repulsing those expressing UNC-5 or UNC-40/UNC-5. Growth cones expressing UNC-40/UNC-5 heterodimers migrate into more dorsal positions when compared to UNC-5 alone. In *unc-6* mutant animals, the lack of UNC-6 gradient prevents dorsoventral migration. Therefore, migrating neurons only travel in the anterior-posterior axis. Students were asked to complete the names of the receptors expressed at the growth cone for each migrating pattern (dashed boxes).

Students were asked to complete the image in Figure 5 and to explain their choices.

## 2. Materials and Methods

### 2.1. List of Materials Necessary for the Experimental Activity

- Stereomicroscope (SZX7, Olympus, Shinjuku, Japan);
- Nematode growth medium (NGM) plates seeded with *Escherichia coli* strain OP50 strain;
- Score tables;
- Fluorescence stereomicroscope (SZX16, Olympus, Shinjuku, Japan) or pre-acquired images of the strains (Figure 4).

### 2.2. C. elegans Strains and Growth Conditions

Standard methods were used for maintaining and culturing *C. elegans* [27]. Nematodes were grown on nematode growth medium (NGM) plates seeded with *Escherichia coli* (*E. coli*) OP50 strain at 20 °C (Table 3).

### 2.3. Generation of Mutant Strains with Fluorescent Nervous System

Males of the quiz strain were generated by mating with N2 (WT) males. To generate double mutant animals, hermaphrodites at the L4 stage of mutant *unc-5* (*e53*), *unc-6* (*e78*), and *unc-40* (*n324*) animals and GFP-positive males were placed on an NGM 30 mm seeded plate at 16 °C for 48 h. Adult hermaphrodites were singled out, and male progeny were evaluated after three days. L4-stage fluorescent animals were singled out, and F2 progeny were selected by UNC phenotypic manifestation and expression of the fluorescent marker (GFP-positive animals). These strains are available upon request.

### 2.4. Microscopy—Stereomicroscope and Video Acquisition

Ten gravid adult hermaphrodite animals of WT, *unc-6*, *unc-5,* and *unc-40* mutant strains were transferred to climatized 60 mm NGM plates seeded with OP50 and allowed to lay eggs for 2 h. After the elimination of the adult animals, eggs were allowed to develop at 20 °C for 4 days. On day 4, plates were renamed mutant A, B, or C and ready for class. For the recording of animals’ motor behavior, the recording system set up comprised a stereomicroscope (SZX7, Olympus), a digital camera (SC30, Olympus), and imaging software (Olympus cellSens Entry 3.2 Software). Recordings were performed in 30 mm seeded NGM plates or liquid media (M9 buffer). A 0.8× zoom lens was used to maximize the field of view. The recording was made for 20 s at a rate of 15 frames per second. Recorded images are shown in real-time.

### 2.5. Confocal Microscopy and Image Building

Four-day-old *unc-6*; GFP, *unc-5*; GFP, *unc-40*; GFP animals, synchronized by egg laying, were immobilized with 3 mM levamisole (Sigma-Aldrich, Missouri, USA) and mounted on a 3% agarose pad. Images of the nervous system of live animals were captured using an Olympus LPS Confocal FV1000 microscope (Confocal microscope, Olympus, FV1000) under a 60× oil (NA = 1.35) objective. Z-series imaging, covering the animal’s total length, was performed using a 488 nm laser excitation line for GFP. The pinhole was adjusted to 1.0 Airy unit of optical slice, and a scan was acquired every ~0.5 μm along the Z-axis. Maximal projection of Z-series images was obtained using Olympus software (FluoView FV1000 Software v2.6, Olympus, Shinjuku, Japan), and images combined using ImageJ (Fiji v1.5) and Adobe Photoshop (CS6, Adobe Systems) software.

### 2.6. Activity Rating

Subjects included the second-year medical students who agreed to participate in the rating of the activity (*n* = 44), which happened right after the end of the class. All students answered eight questions about using *C. elegans* as a model system to understand axonal guidance (4 questions) and the activity (4 questions). 

## 3. Results and Discussion

**Figure 6:**
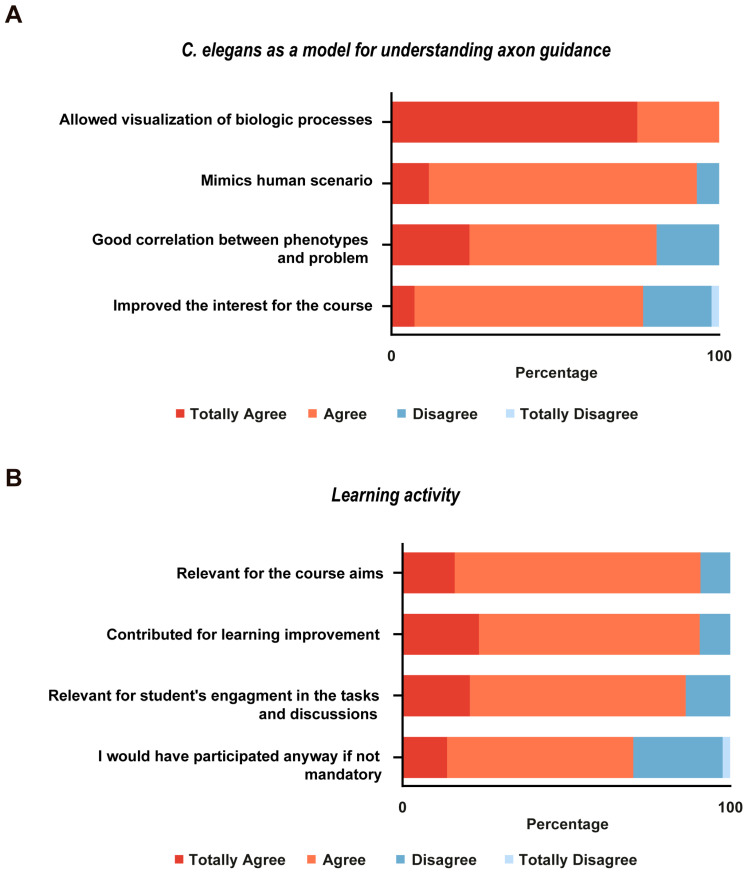
Activity rating by the students. Students were asked to evaluate the activity regarding two parameters: (**A**) the use of *C. elegans* as a model system for understanding the biological phenomenon of axon guidance, and (**B**) its impact on learning, using a Likert 4-points scale (totally disagree, disagree, agree, and totally agree). All students indicated that using *C. elegans* permitted visualization of the phenomena (100%). The majority thought it allowed mimicking the human mechanisms of axonal pathfinding (81.8% agreed and 11.4% agreed totally, while only 6.8% disagreed), and establishing a good correlation between worms’ genotype and phenotype (57.1% agreed, 23.8% agreed totally, and 19.1% disagreed), therefore improving their interest on the course (totally agree 2.3%, agree 69.8%, disagree 20.9%, and totally disagree 2.3%). The majority of the students also highlighted the relevance of the activity for their understanding of the course aims (totally agree 15.9%, agree 75%, and disagree 9.1%), contributing to learning improvement (totally agree 23.3%, agree 67.4%, and disagree 9.3%). Students agreed to be more engaged in the tasks and discussions (totally agree 20.5%, agree 65.9%, and disagree 13.6%) and would have participated in the class, even if not mandatory (totally agree 13.6%, agree 56.8%, disagree 27.3%, and totally disagree 2.3%).

We used this learning activity as part of a nervous system-focused module over 14 years (2004 to 2018), with very positive informal feedback from participants. We applied a formal activity assessment questionnaire in one of the teaching years (*n* = 44). The students’ perception of the activity was gathered with eight questions in a Likert 4-points scale (scores from totally disagree to totally agree), the results of which are shown in Figure 6. This confirmed the students’ satisfaction with the activity and its perceived utility for the acquisition of the core mechanisms underlying axonal pathfinding and the relationship of this process with whole organism structural and behavioral phenotypes. It also shows that the secondary aim of promoting awareness of the relevance of model organisms in biomedical research was achieved. Specifically, regarding using *C. elegans* as a model system to understand axon guidance, nearly all the students agreed that this model allowed the visualization of the biological phenomenon and mimicked the human scenario (100% and 93.2%, respectively). The majority of the students also highlighted that there is a good correlation between phenotypes and the genetic mutation underlying it, and considered that the use of *C. elegans* improved their interest in the course (Figure 6A). Regarding the learning activity, most students considered it relevant to the course objectives, and thought that it contributed to their learning improvement and activity engagement. Notably, 80% of the students indicated they would have participated in the activity even if not mandatory (Figure 6B). 

This activity has the potential to be used for teaching medical students, as we did here, but also biology, biochemistry, or neuroscience students at the undergraduate or graduate levels. The conversion of this activity into a virtual laboratory could be used on its own for online learning or combined with a laboratory activity to reinforce learning.

If taught at the graduate level, the activity can be expanded to additional molecules and molecule types to add diversity and complexity and make each student group have their own project. Concepts, such as the relevance of receptor-receptor interactions, the contribution of adhesion molecules, the role of the extracellular matrix in establishing molecular gradients, and the role of transcriptional regulation and post-translational modifications of the key molecules, can be introduced in these more advanced contexts. This can also be completed using the virtual lab setup if additional components are added.

## Figures and Tables

**Table 1:** Phenotype scoring form for students. After observation, students were given several keywords to phenotypically characterize the mutant animals: uncoordinated (unc) movement, dumpy, coiler, kinker, paralysis, abnormal locomotion tracts, and disorganized body. Students were expected to fill the table according to the annotation appearing in grey. ?—*P_unc-119_*::GFP (quiz strain).

Strain	Observations	Genotype
N2	Sinusoidal (normal) locomotory movement (note worm tracts in the bacteria lawn) Body appearance Body structures: pharynx and intestine	WT
A	unc, abnormal locomotion tract, kinker	ΔA
B	unc, abnormal locomotion tracts, coiler, paralysis (in some animals)	ΔB
C	unc, abnormal locomotion tract, dumpy, disorganized body	ΔC
Quiz	Similar to N2 strain	?

**Table 2:** Nervous system defects form for students. Students were given keywords to characterize the nervous system defects of mutant animals after observation: present, absent, normal, or abnormal. Students were expected to fill the table according to the annotation appearing in grey. VNC—ventral nerve cord, DNC—dorsal nerve cord.

Mutant Animals	Nervous System Phenotype
WT	VNC: present, normal DNC: present, normal Commissures: present, normal Sublateral nerve tracts: present, normal
A = *unc-6* mutant	VNC: present, abnormal DNC: present, abnormal Commissures: absent Sublateral nerve tracts: present, abnormal
B = *unc-5* mutant	VNC: present, normal DNC: absent Commissures: absent Sublateral nerve tracts: present, abnormal
C = *unc-40* mutant	VNC: present, normal DNC: present, normal Commissures: present, abnormal Sublateral nerve tracts: present, abnormal

**Table 3:** *C. elegans* strains used.

Strain	ID	Genotype
N2	Bristol	WT
*unc-6*	CB78	*unc-6* (*e78*) X
*unc-5*	DR169	*che-3* (*e1378*) I; *unc-5* (*e53*) IV
*unc-40*	MT324	*unc-40* (*n324*) I
quiz/GFP	OH441	*otIs45* [*P_unc-119_*::GFP] V
*unc-6*; GFP	MAC113	*otIs45* [*P_unc-119_*::GFP] V; *unc-6* (*e78*) X
*unc-5*; GFP	MAC281	*unc-5* (*e53*) IV; *otIs45* [*P_unc-119_*::GFP] V
*unc-40*; GFP	MAC283	*unc-40* (*n324*) I; *otIs45* [*P_unc-119_*::GFP] V

## Data Availability

No new data were created.

## Supplementary Materials

The following supporting information can be downloaded at: https://www.mdpi.com/article/10.3390/biomedicines11061731/s1, Supplementary Video S1: Videos of wild-type and axonal migration *C. elegans* mutants. WT, *unc-40*, *unc-6* and *unc-5* animals were recorded crawling in solid agar plates (showing a population or single animals) and swimming in a liquid drop. Videos are shown at normal speed.
